# Supplementary material for: Vortex-based soft magnetic composite with ultrastable permeability up to gigahertz frequencies
Source: Nat Commun. 2024 Mar 12;15:2238. doi: 10.1038/s41467-024-46650-9 (PMC10933455; doi:10.1038/s41467-024-46650-9)
Supplement: Supplementary file 1 — Supplementary Information [file 41467_2024_46650_MOESM1_ESM.pdf]

# Supporting Information

## Vortex-Based Soft Magnetic Composite with Ultrastable Permeability up to Gigahertz Frequencies

Guohua Bai, Jiayi Sun, Zhenhua Zhang, Xiaolian Liu, Sateesh Bandaru,Weiwei Liu, Zhong Li, Hongxia Li, Ningning Wang, Xuefeng Zhang*

Corresponding author E-mail: zhang@hdu.edu.cn

**This PDF file includes:**

**Supplementary Figures 1-17**

**Supplementary Tables 1-2**

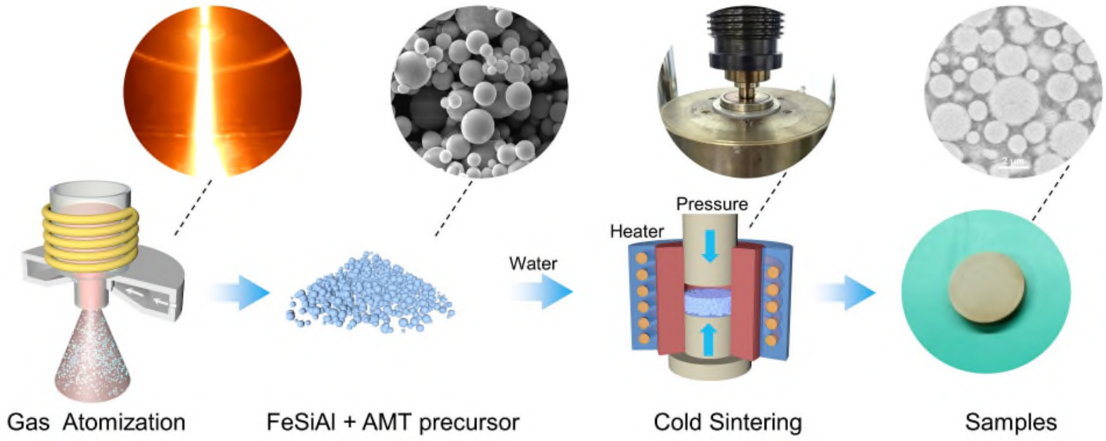


**Supplementary Fig. 1** The schematic of cold sintering process in this work.

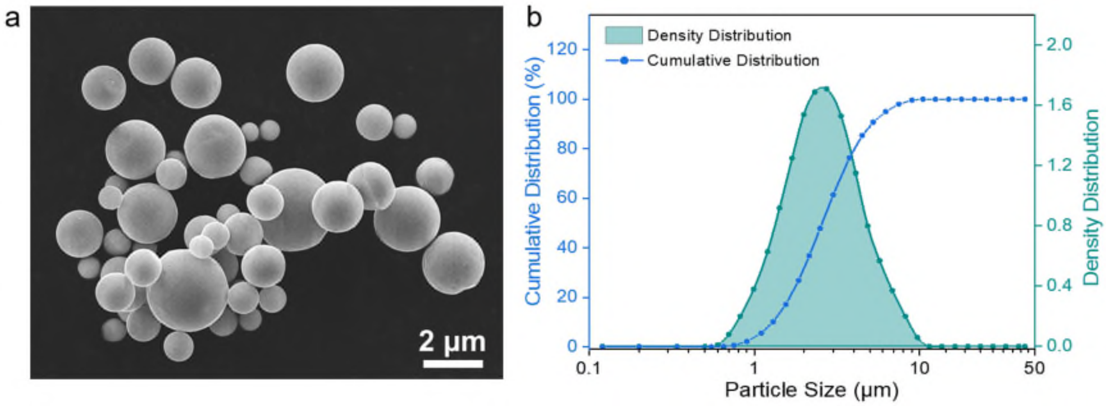


**Supplementary Fig. 2** (a) The morphology and (b) size distribution of ultrafine FeSiAl particles adopted in this work.

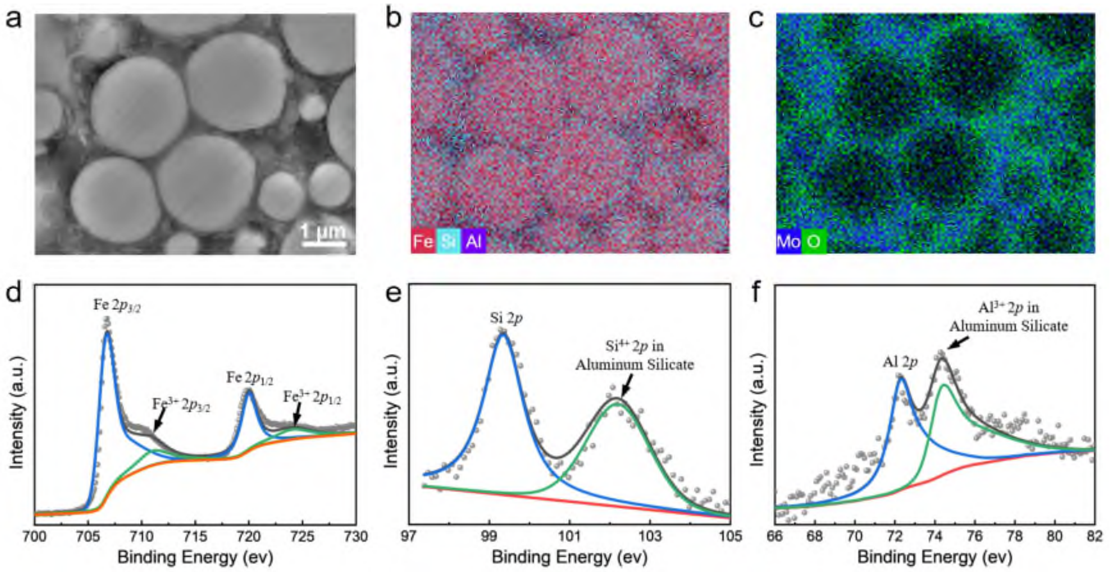


**Supplementary Fig. 3** (a) SEM image and (b, c) EDS elemental mapping, (d-f) XPS spectra of Fe 2*p*, Si 2*p*, and Al 2*p* of CS-AMT&$H_2O$ composite.

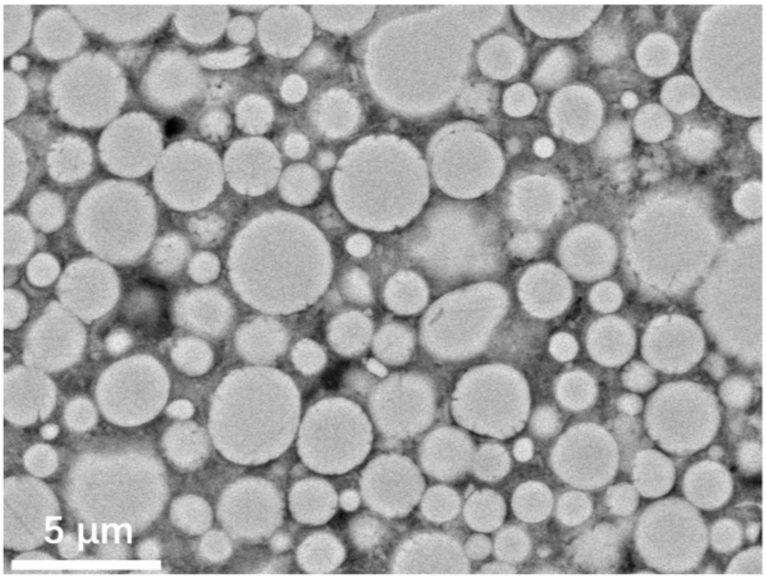


**Supplementary Fig. 4** SEM image of CS-AMT&$H_2O$ composite in a wider view.

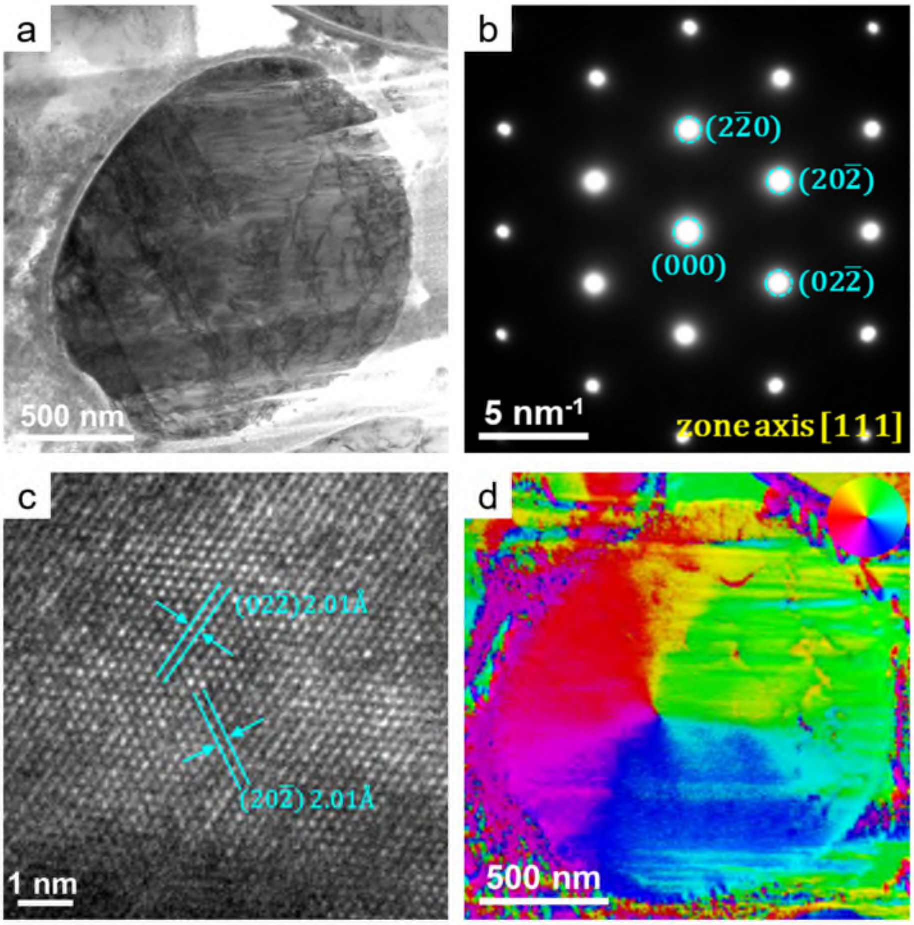


**Supplementary Fig. 5** (a) TEM image of a FeSiAl particle with diameter of 1.6 μm. (b) SAED pattern of the particles, showing a monocrystal structure with zone axis of [111]. (c) HRTEM image with FeSiAl (02$\bar{2}$) and (20$\bar{2}$) planes observed. (d) The DPC image show vortex structure of the particle.

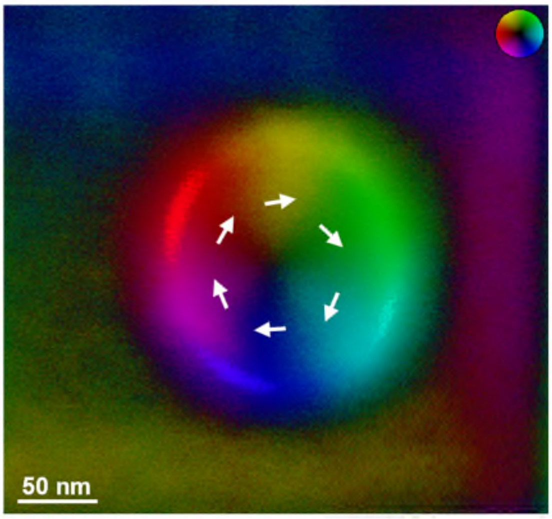


**Supplementary Fig. 6** DPC image of FeSiAl particle with size of 150 nm shows magnetic vortex structure.

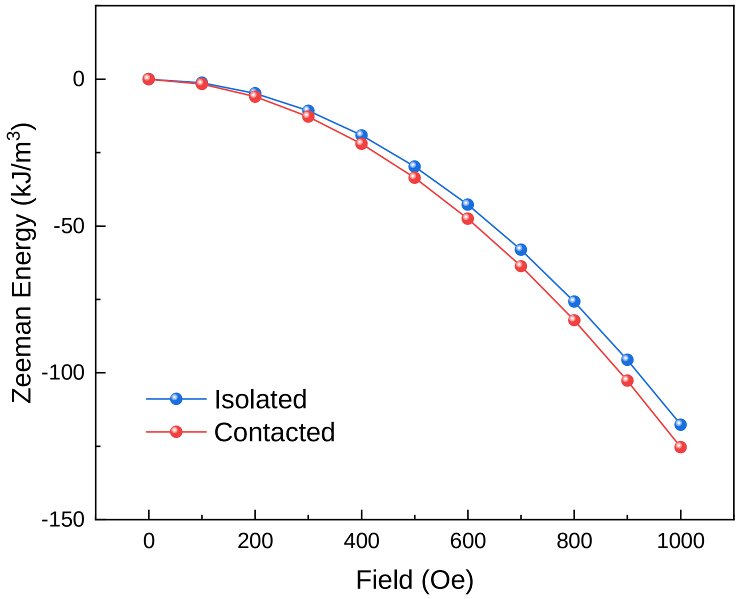


**Supplementary Fig.** 7 The simulated Zeeman energy of isolated and contacted four-vortex assemblies in **Fig. 2**c. Higher Zeeman energy signifies that it is more difficult to align magnetic moment of isolated assembly to external field, which means smaller permeability.

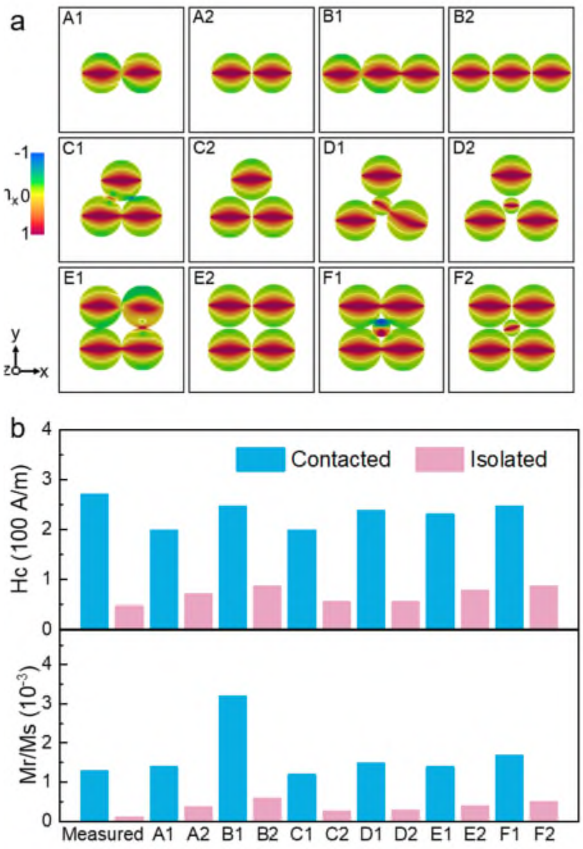


**Supplementary Fig. 8** (a) A1-F1 represent the vortex structure of two, three, four and five contacted particles; A2-F2 represent the vortex structure of two, three, four and five isolated particles. (b) The simulated remanence ($M_r$) and $H_c$ of isolated (red color) and contacted (blue color) particles with different configuration. The measured data corresponds to CS-AMT&$H_2O$ and CS-$H_2O$ composites.

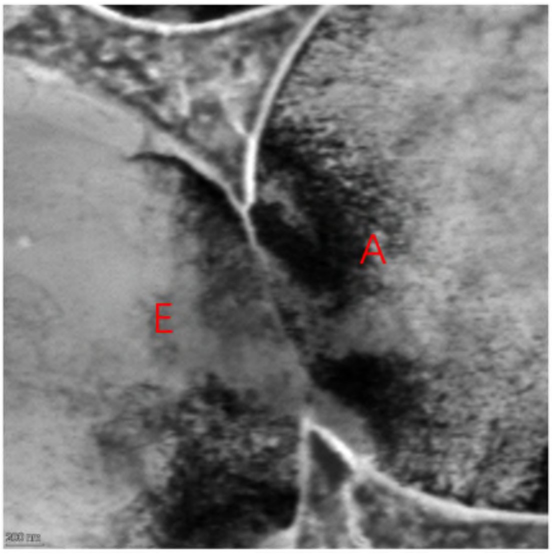


**Supplementary Fig. 9** HAADF image of two contacted particles in **Fig. 2**f.

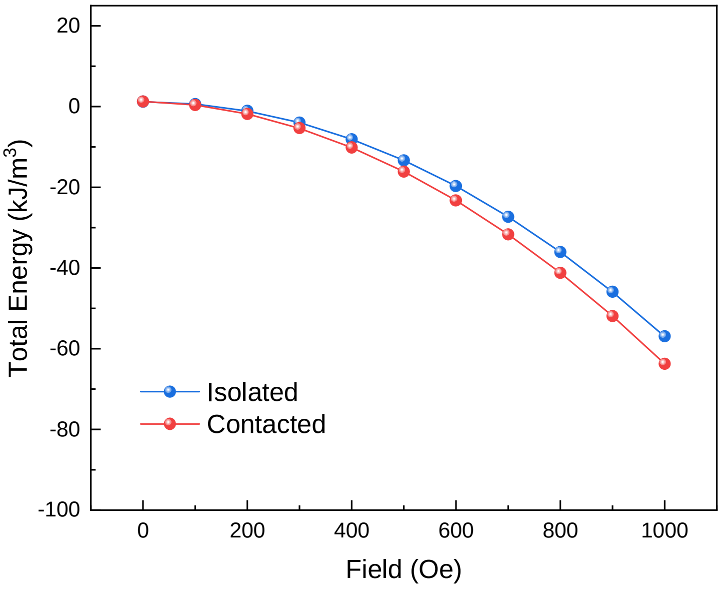


**Supplementary Fig. 10** The simulated total energy of isolated and contacted four-vortex assemblies in **Fig. 2**c.

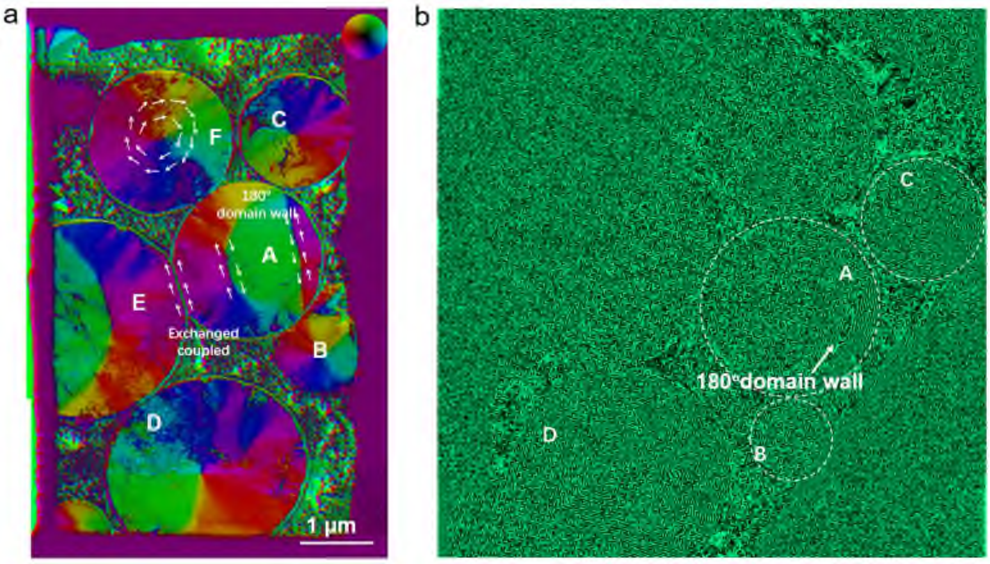


**Supplementary Fig. 11** (a) DPC and (b) corresponding electron holography characterization to confirm the vortex and multidomain structure of FeSiAl particles.

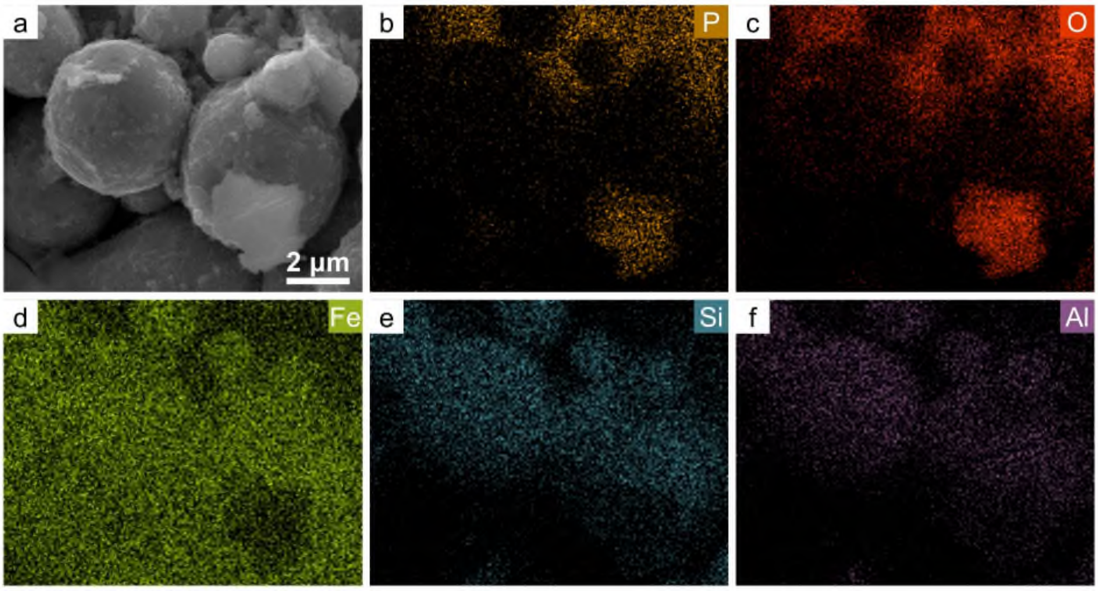


**Supplementary Fig. 12** (a) The fracture surface morphology of cold-pressed CP-PA&SR composite. The integrity of phosphate insulative coating is observed to collapse by the high pressure applied. (b-f) The EDS mapping of Fe, Si, Al, P, O.

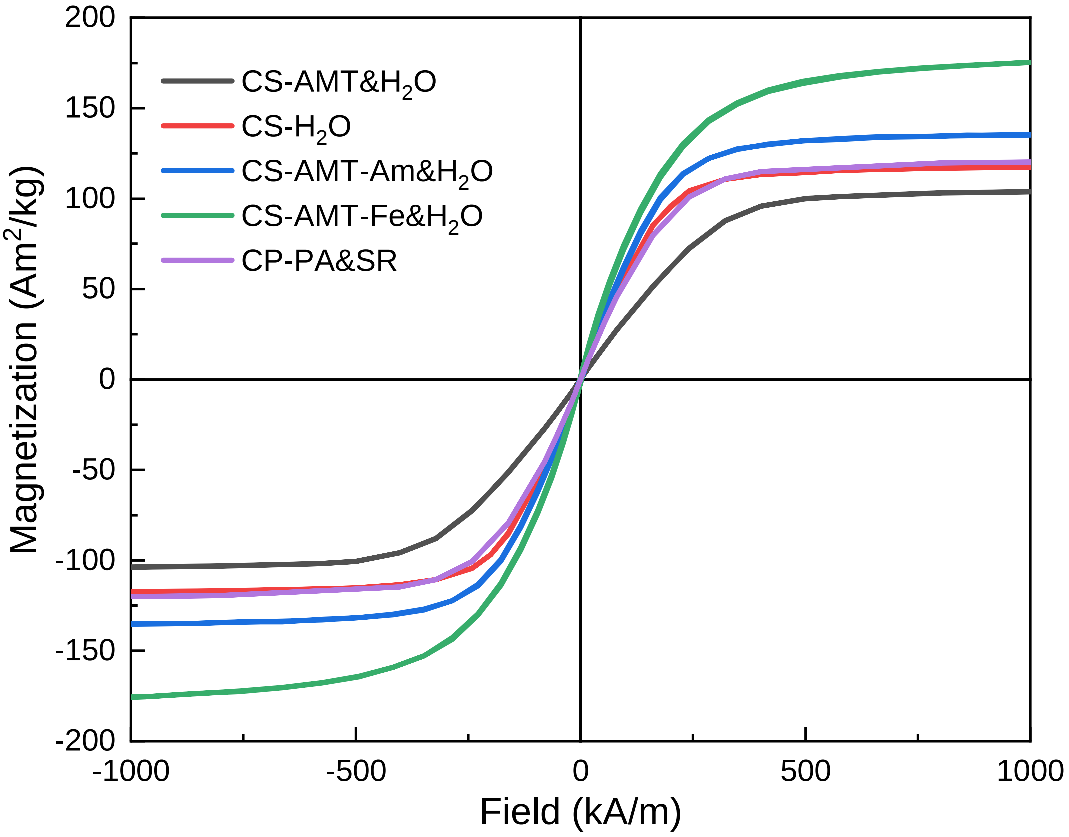


**Supplementary Fig. 13** Hysteresis loops of cold-sintered composites CS-AMT&$H_2O$ and CS-$H_2O$.

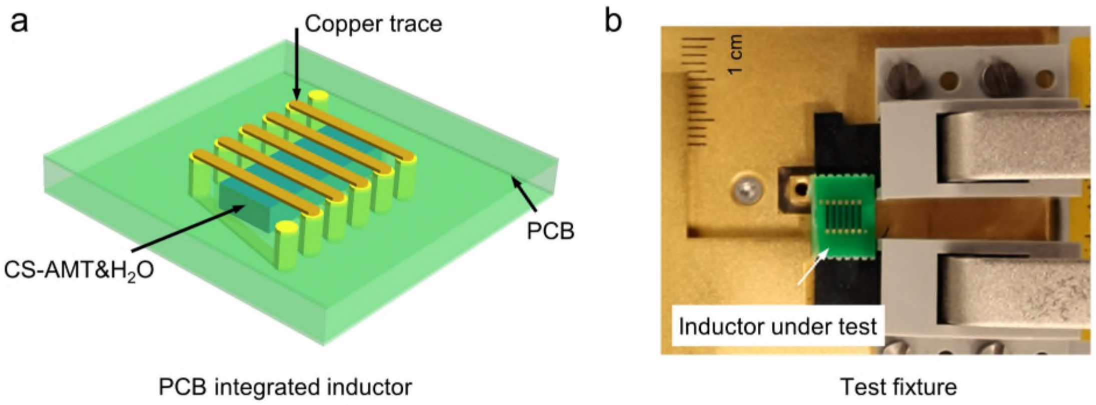


**Supplementary Fig. 14** (a) Sketch and (b) test fixture of PCB-embedded inductor.

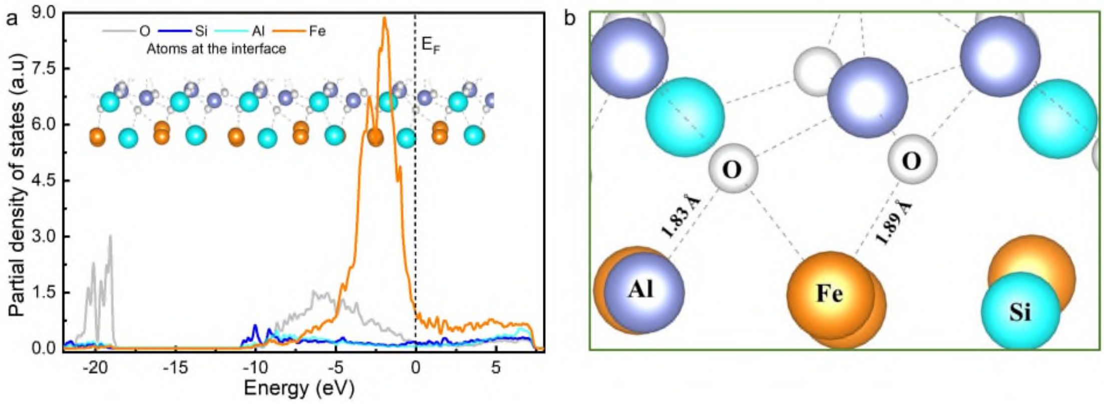


**Supplementary Fig. 15** (a) O, Fe, Al and Si atoms involved partial density of states at the interface. (b) The important interactions between the interfacial atoms and corresponding distances.

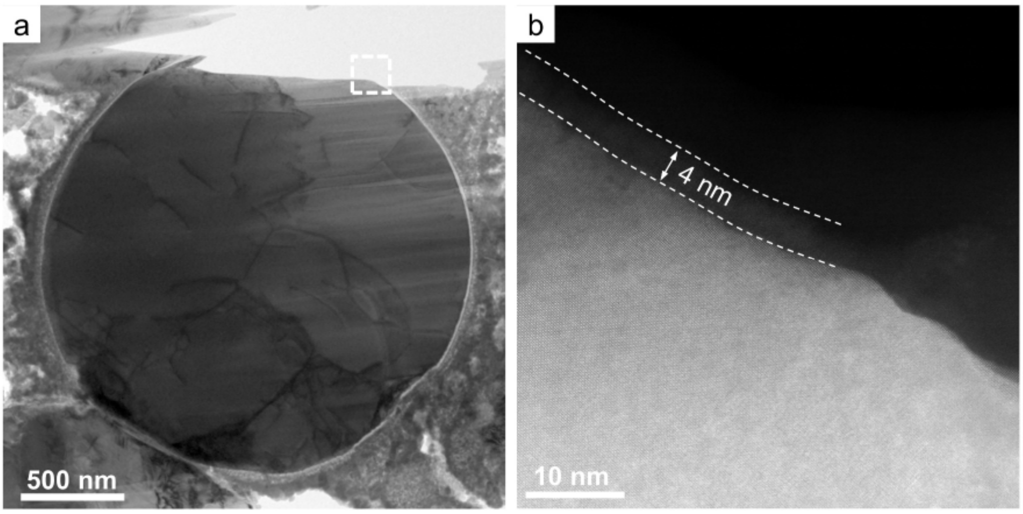


**Supplementary Fig. 16** (a) The white square zone represents the fresh FeSiAl surface produced during FIB process. (b) The STEM image reveals a naturally oxidized amorphous thin layer with a thickness of 4 nm.

**Method to estimate the coercivity of vortex-based composite using simulated coercivity of different particle sizes:**

When simulating the hysteresis loops of parties with different diameter, we find that the slope of all hysteresis is close to 3 (as illustrated in **Fig. R12**). So, the hysteresis loop for particle *i* in the near-zero field region can be approximately expressed as:

$$M_i=3F+M_{ri}$$

Where $M_i$ is the magnetization at a certain external field $F$, and $M_{ri}$ is the remanence of particle *i* obtained from the hysteresis loop.

For composite containing isolated particles with various diameters, its coercivity $H_c$ represents the reversal field at which the sum of magnetization of all particles equals zero, which means:

$$\sum_i f_i M_i = \sum_i f_i (3F + M_{ri}) = \sum_i 3f_i F + \sum_i f_i M_{ri} = 0$$

Where $f_i$ represents the volume fraction of particle $i$. Since $\sum_i f_i = 1$ for a composite, replace $F$ with $H_c$, we have

$$3H_c \sum_i f_i = -\sum_i f_i M_{ri}$$

For particle $i$, its remanence $M_{ri}$ and coercivity $H_{ci}$ is correlated by:

$$M_{ri} = 3H_{ci}$$

So, the coercivity of composite can be expressed as:

$$3H_c = -\sum_i f_i M_{ri} = -\sum_i 3f_i H_{ci}$$

Then we have:

$$H_c = -\sum_i f_i H_{ci}$$

The volume fraction $f_i$ for each particle size is obtained from size distribution measurement. The coercivity $H_{ci}$ for each particle size is obtained for micromagnetic simulation (listed in **supplementary Table 1**).

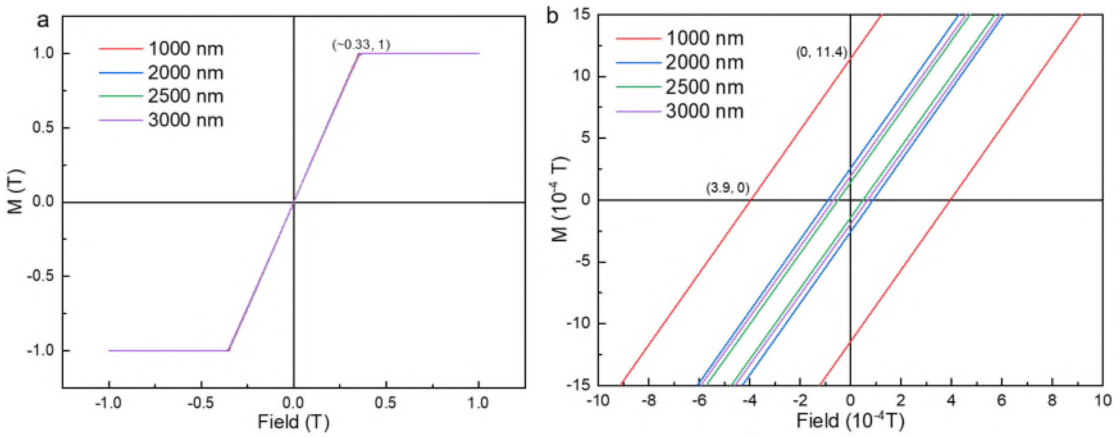


**Supplementary Fig. 17** (a) Typical simulated hysteresis loops for a particle with diameter of 1000 nm, 2000 nm, 2500 nm, 3000 nm. The result shows a linear hysteresis loop with slope of ~3. (b) Enlarged hysteresis loops in the near-zero field region.

**Supplementary Table 1** List of particle diameter, volume fraction and simulated coercivity.

| Particle Diameter Range (μm) | Representative Particle Size (μm) | Volume Fraction (%) | Simulated Coercivity (A/m) |
|---|---|---|---|
| 0.65 | 0.6 | 0.1 | 800 |
| 0.7±0.05 | 0.7 | 0.52 | 664 |
| 0.83±0.07 | 0.8 | 1.56 | 520 |
| 1.0±0.1 | 1 | 3.34 | 320 |
| 1.2±0.1 | 1.2 | 4.6 | 240 |
| 1.43±0.13 | 1.4 | 7.03 | 178.4 |
| 1.7±0.15 | 1.6 | 9.59 | 137.6 |
| 2±0.15 | 2 | 10.02 | 88 |
| 2.33±0.18 | 2.4 | 11.1 | 50.4 |
| 2.75±0.25 | 2.8 | 13.54 | 34.4 |
| 3.37±0.37 | 3.4 | 14.85 | 32 |
| 4.13±0.37 | 4.0 | 9.09 | 49.6 |
| 4.87±0.37 | 5.0 | 5.39 | 11.2 |
| 5.75±0.5 | 5.8 | 4.33 | 32 |
| 6.87±0.52 | 7.0 | 2.97 | 8.8 |
| 8.25±0.75 | 8.0 | 1.88 | 56 |
| 9.75 | 10.0 | 0.39 | 70.4 |

**Supplementary Table 2** The formation energy of different oxides from Al, Si, Mo and Fe.

| Reaction | Formation energy (eV) |
|---|---|
| $2Al + 5/2O_2 + Si = Al_2O_3+SiO_2$ | -23.591eV |
| $2Al + 5/2O_2 + Si = Al_2SiO_5$ | -23.769 eV |
| $2Fe + 3Mo + 6O_2 = Fe_2O_3+3MoO_3$ | -29.508 eV |
| $2Fe + 3Mo + 6O_2 = Fe_2(MoO_4)_3$ | -30.290 eV |
